# Ethyl *N*-(2-benzoyl-3-oxo-3-phenyl­propano­yl)carbamate

**DOI:** 10.1107/S1600536813000445

**Published:** 2013-01-12

**Authors:** Mehmet Akkurt, Ahmet Oral Sarıoğlu, Mehmet Sönmez, Muhammad Nawaz Tahir

**Affiliations:** a Department of Physics, Faculty of Sciences, Erciyes University, 38039 Kayseri, Turkey; b Department of Chemistry, Faculty of Arts and Sciences, Gaziantep University, 27310 Şehitkamil–Gaziantep, Turkey; c Department of Physics, University of Sargodha, Sargodha, Pakistan

## Abstract

In the title compound, C_19_H_17_NO_5_, the dihedral angle between the phenyl groups is 79.55 (15)°. The terminal eth­oxy group is disordered over two orientations in a 0.873 (6):0.127 (6) ratio. In the crystal, mol­ecules are linked by N—H⋯O and C—H⋯O hydrogen bonds into [001] chains which incorporate *R*_1_^2^(6) loops. A very weak C—H⋯π contact also occurs.

## Related literature

For background to the carboxamide [–C(O)NH–] group, see: Sönmez (2001 ▶). For further synthetic details, see: Fabian *et al.* (1992 ▶).
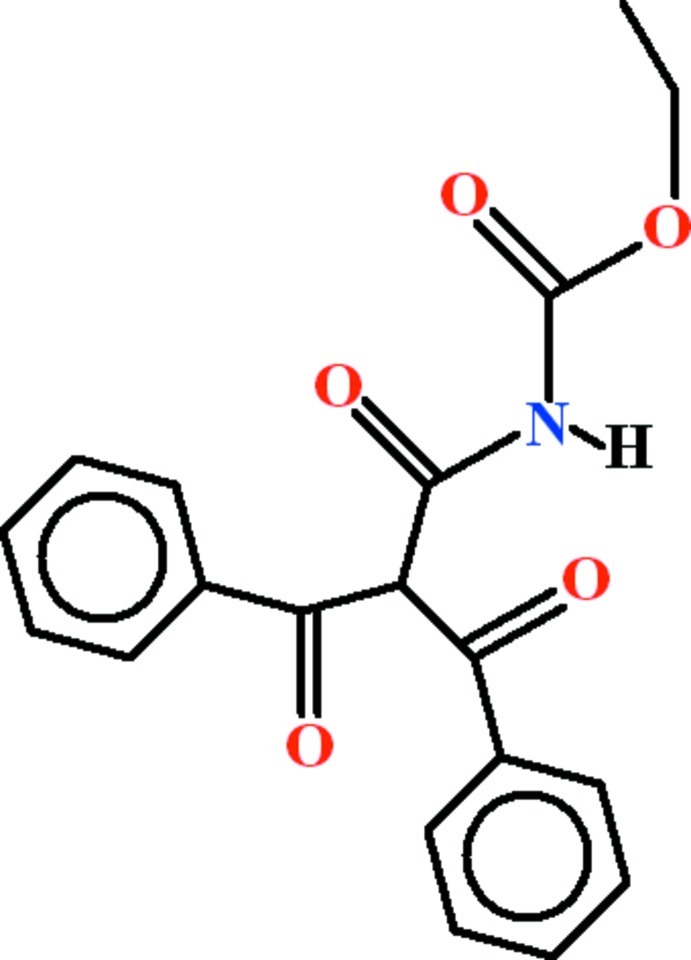


## Experimental

### 

#### Crystal data

C_19_H_17_NO_5_
*M* *_r_* = 339.34
Monoclinic, 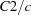

*a* = 33.088 (8) Å
*b* = 12.732 (3) Å
*c* = 8.7110 (18) Å
β = 97.896 (9)°
*V* = 3635.0 (14) Å^3^
*Z* = 8
Mo *K*α radiation
μ = 0.09 mm^−1^
*T* = 296 K
0.35 × 0.18 × 0.16 mm

#### Data collection

Bruker Kappa APEXII CCD diffractometer
Absorption correction: multi-scan (*SADABS*; Bruker, 2009 ▶) *T*_min_ = 0.981, *T*_max_ = 0.986
14531 measured reflections
3579 independent reflections
1910 reflections with *I* > 2σ(*I*)
*R*_int_ = 0.050

#### Refinement

*R*[*F* ^2^ > 2σ(*F* ^2^)] = 0.049
*wR*(*F* ^2^) = 0.132
*S* = 1.01
3579 reflections
234 parameters
4 restraints
H-atom parameters constrained
Δρ_max_ = 0.16 e Å^−3^
Δρ_min_ = −0.13 e Å^−3^

### 

Data collection: *APEX2* (Bruker, 2009 ▶); cell refinement: *SAINT* (Bruker, 2009 ▶); data reduction: *SAINT*; program(s) used to solve structure: *SHELXS97* (Sheldrick, 2008 ▶); program(s) used to refine structure: *SHELXL97* (Sheldrick, 2008 ▶); molecular graphics: *ORTEP-3* (Farrugia, 2012 ▶) and *PLATON* (Spek, 2009 ▶); software used to prepare material for publication: *WinGX* (Farrugia, 2012 ▶) and *PLATON*.

## Supplementary Material

Crystal structure: contains datablock(s) global, I. DOI: 10.1107/S1600536813000445/hb7023sup1.cif

Structure factors: contains datablock(s) I. DOI: 10.1107/S1600536813000445/hb7023Isup2.hkl

Supplementary material file. DOI: 10.1107/S1600536813000445/hb7023Isup3.cml

Additional supplementary materials:  crystallographic information; 3D view; checkCIF report

## Figures and Tables

**Table 1:** Hydrogen-bond geometry (Å, °) *Cg*2 is the centroid of the C10–C15 phenyl ring.

*D*—H⋯*A*	*D*—H	H⋯*A*	*D*⋯*A*	*D*—H⋯*A*
N1—H1⋯O3^i^	0.86	2.37	3.025 (2)	133
N1—H1⋯O4^i^	0.86	2.08	2.842 (2)	147
C8—H8⋯O3^i^	0.98	2.38	3.263 (3)	150
C19*B*—H19*F*⋯*Cg*2^ii^	0.96	2.96	3.786 (5)	145

Symmetry codes: (i) 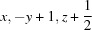; (ii) 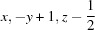.

**Fig. 1.**
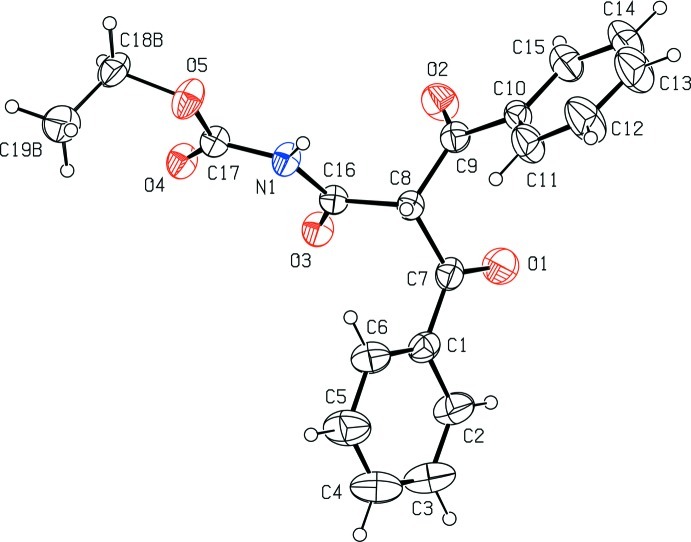
The molecular structure of (I) with displacement ellipsoids for non-H atoms drawn at the 30% probability level. Only the major disorder component is shown.

**Fig. 2.**
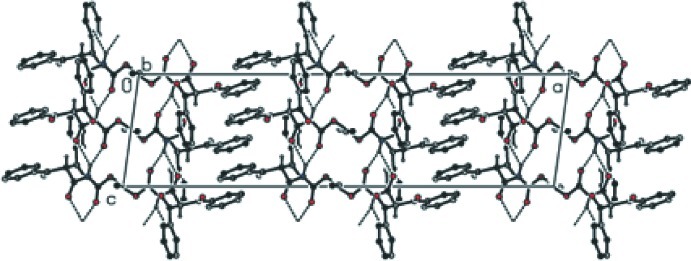
The packing and hydrogen bonding of the title compound, viewing along the b axis. H atoms not involved in hydrogen bonding and the minor disordered component are omitted for clarity.

## supplementary crystallographic information

### Comment

The carboxamide [–C(O)NH–] group, which seems to be everywhere throughout nature in the primary structure of proteins, is an important ligand construction unit for coordination chemists (Sönmez, 2001). The high stability of the amide linkage toward hydrolysis is of crucial importance to biological systems, since it allows the construction of peptides from relatively simple amino acid precursors.

In the title compound (I), (Fig. 1), the C1–C6 and C10–C15 phenyl rings make a dihedral angle of 79.55 (15)° with each other. The C7–C8–C16–O3, C8–C16–N1–C17, O3–C16–N1–C17, C16–N1–C17–O4 and C16–N1–C17–O5 torsion angles are -23.0 (3), -176.6 (2), 3.9 (4), 2.6 (4) and -177.2 (2)°, respectively.

In the crystal structure, N—H···O and C—H···O hydrogen bonds (Table 1, Fig. 2) connect the neighbouring molecules, into chains running along the *c* axis, forming the *R*^2^_1_(6) motifs (Fig. 2). Furthermore, C—H···π interactions between the H19F hydrogen atom of the methyl group and the C10–C15 phenyl ring (with centroid *Cg*2) is also observed (Table 1).

### Experimental

Dibenzoylaceticacid-*N*-carboxyethylamide was prepared from reaction of 4-benzoyl-5-phenyl-2,3-furandione and ethyl urethane as the method reported earlier (Fabian *et al.*, 1992). These compounds were refluxed in benzene for 5 h. The solvent was evaporated under reduced pressure to give an oily residue which was treated with ether and finally crystallized from absolute ethanol as colourless needles. Analysis calculated for (C_19_H_17_NO_5_): C 67.25, H 5.01, N 4.14. Found: C 67.22, H 5.06, N 4.30.

### Refinement

All H atoms were positioned geometrically and refined by using a riding model, with N—H = 0.86 Å (amine), C—H = 0.93 (aromatic), C—H = 0.96 (methyl), C—H = 0.97 (methylene) and 0.98 Å (methine), and *U*_iso_(H) = 1.2 or 1.5*U*_eq_(C,*N*). The C atoms of the terminal ethoxy group are disordered over two positions with occupancy ratio 0.873 (6):0.127 (6). The temperature factors of the disordered C atoms were refined with the EADP restraint.

The unit cell contains a pair of voids of 44 (2)Å^3^ volume located about an inversion centre but the residual electron density (highest peak = 0.160 e Å^-3^and deepest hole = -0.126 e Å^-3^) in the difference Fourier map suggests that no solvent molecule occupies this void.

### Crystal data

**Table:**

C_19_H_17_NO_5_	*F*(000) = 1424
*M**_r_* = 339.34	*D*_x_ = 1.240 Mg m^−^^3^
Monoclinic, *C*2/*c*	Mo *K*α radiation, λ = 0.71073 Å
Hall symbol: -C 2yc	Cell parameters from 270 reflections
*a* = 33.088 (8) Å	θ = 3.1–21.4°
*b* = 12.732 (3) Å	µ = 0.09 mm^−^^1^
*c* = 8.7110 (18) Å	*T* = 296 K
β = 97.896 (9)°	Needle, white
*V* = 3635.0 (14) Å^3^	0.35 × 0.18 × 0.16 mm
*Z* = 8	

### Data collection

**Table:**

Bruker Kappa APEXII CCD diffractometer	3579 independent reflections
Radiation source: fine-focus sealed tube	1910 reflections with *I* > 2σ(*I*)
Graphite monochromator	*R*_int_ = 0.050
ω scans	θ_max_ = 26.0°, θ_min_ = 1.2°
Absorption correction: multi-scan (*SADABS*; Bruker, 2009)	*h* = −37→40
*T*_min_ = 0.981, *T*_max_ = 0.986	*k* = −15→15
14531 measured reflections	*l* = −8→10

### Refinement

**Table:**

Refinement on *F*^2^	Secondary atom site location: difference Fourier map
Least-squares matrix: full	Hydrogen site location: inferred from neighbouring sites
*R*[*F*^2^ > 2σ(*F*^2^)] = 0.049	H-atom parameters constrained
*wR*(*F*^2^) = 0.132	*w* = 1/[σ^2^(*F*_o_^2^) + (0.0486*P*)^2^ + 0.910*P*] where *P* = (*F*_o_^2^ + 2*F*_c_^2^)/3
*S* = 1.01	(Δ/σ)_max_ < 0.001
3579 reflections	Δρ_max_ = 0.16 e Å^−^^3^
234 parameters	Δρ_min_ = −0.13 e Å^−^^3^
4 restraints	Extinction correction: *SHELXL97* (Sheldrick, 2008), FC^*^=KFC[1+0.001XFC^2^Λ^3^/SIN(2Θ)]^-1/4^
Primary atom site location: structure-invariant direct methods	Extinction coefficient: 0.0030 (4)

### Special details

**Table:**

Geometry. Bond distances, angles *etc*. have been calculated using the rounded fractional coordinates. All su's are estimated from the variances of the (full) variance-covariance matrix. The cell e.s.d.'s are taken into account in the estimation of distances, angles and torsion angles
Refinement. Refinement on *F*^2^ for ALL reflections except those flagged by the user for potential systematic errors. Weighted *R*-factors *wR* and all goodnesses of fit *S* are based on *F*^2^, conventional *R*-factors *R* are based on *F*, with *F* set to zero for negative *F*^2^. The observed criterion of *F*^2^ > σ(*F*^2^) is used only for calculating -*R*-factor-obs *etc*. and is not relevant to the choice of reflections for refinement. *R*-factors based on *F*^2^ are statistically about twice as large as those based on *F*, and *R*-factors based on ALL data will be even larger.

### Fractional atomic coordinates and isotropic or equivalent isotropic displacement parameters (Å^2^)

**Table:**

	*x*	*y*	*z*	*U*_iso_*/*U*_eq_	Occ. (<1)
O1	0.18768 (6)	0.74420 (16)	0.0969 (2)	0.0913 (9)	
O2	0.09155 (6)	0.76202 (14)	0.08336 (19)	0.0757 (8)	
O3	0.12375 (5)	0.56205 (12)	−0.09053 (15)	0.0564 (6)	
O4	0.05773 (5)	0.42206 (13)	−0.13912 (16)	0.0578 (6)	
O5	0.03265 (5)	0.39586 (14)	0.08394 (17)	0.0693 (7)	
N1	0.08534 (6)	0.50073 (15)	0.08736 (18)	0.0494 (7)	
C1	0.21155 (8)	0.5710 (2)	0.1313 (2)	0.0558 (10)	
C2	0.24785 (9)	0.5949 (3)	0.0759 (3)	0.0819 (12)	
C3	0.27677 (11)	0.5189 (4)	0.0679 (4)	0.1085 (18)	
C4	0.27046 (12)	0.4194 (3)	0.1159 (4)	0.1102 (17)	
C5	0.23524 (12)	0.3949 (3)	0.1725 (4)	0.0984 (17)	
C6	0.20565 (9)	0.4704 (2)	0.1804 (3)	0.0707 (11)	
C7	0.18068 (8)	0.6541 (2)	0.1304 (3)	0.0553 (10)	
C8	0.13836 (7)	0.62652 (16)	0.1695 (2)	0.0435 (8)	
C9	0.11479 (8)	0.72705 (18)	0.1908 (3)	0.0511 (9)	
C10	0.12157 (8)	0.78140 (18)	0.3436 (3)	0.0520 (9)	
C11	0.14062 (9)	0.7351 (2)	0.4765 (3)	0.0754 (11)	
C12	0.14478 (11)	0.7874 (3)	0.6156 (3)	0.1070 (18)	
C13	0.13103 (12)	0.8877 (3)	0.6216 (4)	0.1085 (18)	
C14	0.11213 (11)	0.9365 (2)	0.4910 (4)	0.0946 (14)	
C15	0.10659 (9)	0.8823 (2)	0.3516 (3)	0.0717 (11)	
C16	0.11539 (7)	0.56060 (17)	0.0400 (2)	0.0444 (8)	
C17	0.05827 (7)	0.43737 (18)	−0.0035 (2)	0.0475 (8)	
C18B	−0.00031 (11)	0.3297 (3)	0.0107 (4)	0.0681 (16)	0.874 (6)
C19B	0.01476 (14)	0.2202 (3)	0.0081 (6)	0.122 (2)	0.874 (6)
C19A	−0.0059 (10)	0.230 (2)	0.104 (4)	0.122 (2)	0.127 (6)
C18A	0.0143 (10)	0.302 (2)	−0.003 (3)	0.0681 (16)	0.127 (6)
H2	0.25255	0.66297	0.04389	0.0984*	
H1	0.08306	0.50283	0.18448	0.0593*	
H5	0.23111	0.32685	0.20608	0.1180*	
H6	0.18166	0.45305	0.21893	0.0846*	
H8	0.14126	0.58613	0.26611	0.0522*	
H11	0.15087	0.66726	0.47217	0.0903*	
H12	0.15696	0.75432	0.70541	0.1281*	
H13	0.13449	0.92367	0.71546	0.1301*	
H14	0.10307	1.00540	0.49576	0.1135*	
H15	0.09279	0.91396	0.26339	0.0861*	
H18C	−0.00918	0.35382	−0.09419	0.0818*	0.874 (6)
H18D	−0.02337	0.33326	0.06829	0.0818*	0.874 (6)
H19D	−0.00633	0.17611	−0.04401	0.1830*	0.874 (6)
H19E	0.02221	0.19582	0.11238	0.1830*	0.874 (6)
H19F	0.03817	0.21784	−0.04587	0.1830*	0.874 (6)
H3	0.30085	0.53546	0.02943	0.1298*	
H4	0.29018	0.36801	0.11008	0.1319*	
H18A	0.03551	0.26331	−0.04608	0.0818*	0.127 (6)
H18B	−0.00573	0.32448	−0.08842	0.0818*	0.127 (6)
H19A	−0.02677	0.18932	0.04337	0.1830*	0.127 (6)
H19B	−0.01783	0.27177	0.17740	0.1830*	0.127 (6)
H19C	0.01418	0.18378	0.15742	0.1830*	0.127 (6)

### Atomic displacement parameters (Å^2^)

**Table:**

	*U*^11^	*U*^22^	*U*^33^	*U*^12^	*U*^13^	*U*^23^
O1	0.0800 (16)	0.0636 (13)	0.1396 (18)	−0.0162 (11)	0.0482 (13)	−0.0004 (12)
O2	0.0850 (15)	0.0718 (13)	0.0665 (12)	0.0153 (10)	−0.0035 (10)	−0.0004 (9)
O3	0.0666 (12)	0.0714 (11)	0.0345 (8)	−0.0136 (9)	0.0190 (7)	−0.0040 (7)
O4	0.0639 (12)	0.0765 (12)	0.0335 (9)	−0.0142 (9)	0.0081 (7)	−0.0070 (7)
O5	0.0696 (13)	0.0926 (13)	0.0477 (9)	−0.0354 (10)	0.0153 (8)	0.0001 (8)
N1	0.0573 (14)	0.0659 (13)	0.0265 (9)	−0.0173 (10)	0.0107 (8)	−0.0042 (8)
C1	0.0473 (18)	0.0715 (19)	0.0478 (14)	−0.0040 (14)	0.0034 (11)	−0.0022 (12)
C2	0.056 (2)	0.095 (2)	0.097 (2)	0.0000 (18)	0.0189 (16)	0.0082 (17)
C3	0.059 (2)	0.146 (4)	0.125 (3)	0.022 (2)	0.029 (2)	0.017 (3)
C4	0.077 (3)	0.127 (3)	0.127 (3)	0.045 (2)	0.015 (2)	0.017 (2)
C5	0.083 (3)	0.097 (3)	0.115 (3)	0.028 (2)	0.013 (2)	0.026 (2)
C6	0.058 (2)	0.081 (2)	0.0727 (18)	0.0107 (16)	0.0079 (13)	0.0116 (15)
C7	0.0559 (19)	0.0575 (16)	0.0534 (14)	−0.0127 (14)	0.0111 (12)	−0.0060 (12)
C8	0.0475 (16)	0.0496 (14)	0.0339 (11)	−0.0052 (11)	0.0075 (9)	−0.0025 (9)
C9	0.0540 (18)	0.0521 (15)	0.0488 (14)	−0.0059 (12)	0.0131 (12)	0.0011 (11)
C10	0.0574 (17)	0.0474 (15)	0.0547 (14)	−0.0077 (12)	0.0205 (12)	−0.0095 (11)
C11	0.096 (2)	0.0686 (18)	0.0586 (17)	0.0088 (16)	0.0003 (15)	−0.0192 (14)
C12	0.149 (4)	0.102 (3)	0.065 (2)	0.027 (2)	−0.0033 (19)	−0.0311 (18)
C13	0.142 (4)	0.104 (3)	0.079 (2)	0.011 (2)	0.013 (2)	−0.041 (2)
C14	0.129 (3)	0.064 (2)	0.098 (2)	0.0025 (19)	0.041 (2)	−0.0261 (18)
C15	0.091 (2)	0.0568 (18)	0.0722 (18)	−0.0022 (15)	0.0287 (15)	−0.0056 (13)
C16	0.0494 (16)	0.0486 (14)	0.0363 (12)	−0.0022 (11)	0.0102 (10)	0.0017 (10)
C17	0.0522 (17)	0.0552 (14)	0.0357 (12)	−0.0056 (12)	0.0080 (10)	0.0028 (10)
C18B	0.050 (3)	0.079 (3)	0.076 (2)	−0.020 (2)	0.0109 (19)	−0.0027 (16)
C19B	0.079 (3)	0.079 (3)	0.211 (6)	−0.007 (2)	0.032 (3)	−0.025 (3)
C19A	0.079 (3)	0.079 (3)	0.211 (6)	−0.007 (2)	0.032 (3)	−0.025 (3)
C18A	0.050 (3)	0.079 (3)	0.076 (2)	−0.020 (2)	0.0109 (19)	−0.0027 (16)

### Geometric parameters (Å, º)

**Table:**

O1—C7	1.214 (3)	C13—C14	1.370 (5)
O2—C9	1.212 (3)	C14—C15	1.387 (4)
O3—C16	1.207 (2)	C18A—C19A	1.53 (4)
O4—C17	1.195 (2)	C18B—C19B	1.482 (5)
O5—C17	1.325 (3)	C2—H2	0.9300
O5—C18B	1.454 (4)	C3—H3	0.9300
O5—C18A	1.50 (3)	C4—H4	0.9300
N1—C16	1.361 (3)	C5—H5	0.9300
N1—C17	1.373 (3)	C6—H6	0.9300
N1—H1	0.8600	C8—H8	0.9800
C1—C2	1.388 (4)	C11—H11	0.9300
C1—C7	1.470 (4)	C12—H12	0.9300
C1—C6	1.373 (4)	C13—H13	0.9300
C2—C3	1.369 (6)	C14—H14	0.9300
C3—C4	1.359 (6)	C15—H15	0.9300
C4—C5	1.363 (6)	C18A—H18B	0.9700
C5—C6	1.381 (5)	C18A—H18A	0.9700
C7—C8	1.527 (4)	C18B—H18D	0.9700
C8—C9	1.523 (3)	C18B—H18C	0.9700
C8—C16	1.523 (3)	C19A—H19A	0.9600
C9—C10	1.490 (4)	C19A—H19C	0.9600
C10—C11	1.373 (4)	C19A—H19B	0.9600
C10—C15	1.382 (4)	C19B—H19E	0.9600
C11—C12	1.373 (4)	C19B—H19F	0.9600
C12—C13	1.359 (5)	C19B—H19D	0.9600
			
C17—O5—C18B	118.64 (19)	C3—C4—H4	120.00
C17—O5—C18A	105.9 (11)	C5—C4—H4	120.00
C16—N1—C17	126.89 (16)	C4—C5—H5	120.00
C17—N1—H1	117.00	C6—C5—H5	120.00
C16—N1—H1	117.00	C1—C6—H6	120.00
C2—C1—C7	118.3 (3)	C5—C6—H6	120.00
C2—C1—C6	118.7 (3)	C7—C8—H8	109.00
C6—C1—C7	123.0 (2)	C9—C8—H8	109.00
C1—C2—C3	120.5 (3)	C16—C8—H8	109.00
C2—C3—C4	120.3 (3)	C10—C11—H11	119.00
C3—C4—C5	120.0 (4)	C12—C11—H11	120.00
C4—C5—C6	120.4 (3)	C11—C12—H12	120.00
C1—C6—C5	120.1 (3)	C13—C12—H12	120.00
C1—C7—C8	119.4 (2)	C12—C13—H13	120.00
O1—C7—C1	121.8 (2)	C14—C13—H13	120.00
O1—C7—C8	118.8 (2)	C13—C14—H14	120.00
C9—C8—C16	109.97 (19)	C15—C14—H14	120.00
C7—C8—C9	109.54 (19)	C10—C15—H15	120.00
C7—C8—C16	109.95 (17)	C14—C15—H15	120.00
O2—C9—C8	119.7 (2)	H18A—C18A—H18B	108.00
O2—C9—C10	121.4 (2)	O5—C18A—H18A	110.00
C8—C9—C10	118.8 (2)	O5—C18A—H18B	110.00
C9—C10—C11	123.3 (2)	C19A—C18A—H18A	109.00
C11—C10—C15	118.9 (2)	C19A—C18A—H18B	110.00
C9—C10—C15	117.8 (2)	C19B—C18B—H18C	110.00
C10—C11—C12	121.0 (3)	C19B—C18B—H18D	110.00
C11—C12—C13	119.8 (3)	O5—C18B—H18D	110.00
C12—C13—C14	120.8 (3)	O5—C18B—H18C	110.00
C13—C14—C15	119.5 (3)	H18C—C18B—H18D	108.00
C10—C15—C14	120.1 (3)	C18A—C19A—H19A	109.00
O3—C16—N1	124.45 (19)	C18A—C19A—H19B	109.00
N1—C16—C8	113.28 (16)	H19A—C19A—H19C	109.00
O3—C16—C8	122.3 (2)	H19B—C19A—H19C	110.00
O4—C17—N1	125.9 (2)	C18A—C19A—H19C	109.00
O4—C17—O5	125.4 (2)	H19A—C19A—H19B	110.00
O5—C17—N1	108.74 (16)	C18B—C19B—H19F	109.00
O5—C18A—C19A	111 (2)	H19E—C19B—H19F	109.00
O5—C18B—C19B	108.5 (3)	H19D—C19B—H19E	110.00
C1—C2—H2	120.00	H19D—C19B—H19F	109.00
C3—C2—H2	120.00	C18B—C19B—H19D	109.00
C2—C3—H3	120.00	C18B—C19B—H19E	109.00
C4—C3—H3	120.00		
			
C18B—O5—C17—O4	−3.0 (4)	C1—C7—C8—C16	−69.7 (3)
C18B—O5—C17—N1	176.9 (2)	C7—C8—C9—O2	95.7 (3)
C17—O5—C18B—C19B	88.0 (3)	C7—C8—C9—C10	−83.1 (3)
C16—N1—C17—O4	2.7 (4)	C16—C8—C9—O2	−25.2 (3)
C16—N1—C17—O5	−177.2 (2)	C16—C8—C9—C10	156.0 (2)
C17—N1—C16—O3	−3.9 (4)	C7—C8—C16—O3	−23.0 (3)
C17—N1—C16—C8	176.6 (2)	C9—C8—C16—N1	−82.8 (2)
C6—C1—C2—C3	1.2 (4)	C7—C8—C16—N1	156.55 (19)
C7—C1—C2—C3	−177.1 (3)	C9—C8—C16—O3	97.7 (3)
C2—C1—C6—C5	−0.8 (4)	C8—C9—C10—C15	167.1 (2)
C7—C1—C6—C5	177.4 (3)	O2—C9—C10—C11	166.1 (3)
C2—C1—C7—O1	−7.1 (4)	O2—C9—C10—C15	−11.7 (4)
C6—C1—C7—O1	174.7 (2)	C8—C9—C10—C11	−15.1 (4)
C6—C1—C7—C8	−6.5 (3)	C9—C10—C11—C12	−177.6 (3)
C2—C1—C7—C8	171.7 (2)	C15—C10—C11—C12	0.2 (4)
C1—C2—C3—C4	−0.8 (5)	C9—C10—C15—C14	−179.9 (3)
C2—C3—C4—C5	−0.1 (5)	C11—C10—C15—C14	2.2 (4)
C3—C4—C5—C6	0.5 (5)	C10—C11—C12—C13	−2.1 (5)
C4—C5—C6—C1	−0.1 (5)	C11—C12—C13—C14	1.6 (6)
O1—C7—C8—C16	109.2 (2)	C12—C13—C14—C15	0.8 (6)
O1—C7—C8—C9	−11.8 (3)	C13—C14—C15—C10	−2.7 (5)
C1—C7—C8—C9	169.4 (2)		

### Hydrogen-bond geometry (Å, º)

Cg2 is the centroid of the C10–C15 phenyl ring.

**Table:**

*D*—H···*A*	*D*—H	H···*A*	*D*···*A*	*D*—H···*A*
N1—H1···O3^i^	0.86	2.37	3.025 (2)	133
N1—H1···O4^i^	0.86	2.08	2.842 (2)	147
C8—H8···O3^i^	0.98	2.38	3.263 (3)	150
C19*B*—H19*F*···*Cg*2^ii^	0.96	2.96	3.786 (5)	145

Symmetry codes: (i) *x*, −*y*+1, *z*+1/2; (ii) *x*, −*y*+1, *z*−1/2.
